# Image Data-Centric Visual Feature Selection on Roll-to-Roll Slot-Die Coating Systems for Edge Wave Coating Defect Detection

**DOI:** 10.3390/polym16081156

**Published:** 2024-04-19

**Authors:** Yoonjae Lee, Junyoung Yun, Sangbin Lee, Changwoo Lee

**Affiliations:** 1 Department of Mechanical Design and Production Engineering, Konkuk University, 120 Neungdong-ro, Gwangjin-gu, Seoul 05029, Republic of Korea; dldbswp913@konkuk.ac.kr (Y.L.); jun980520@konkuk.ac.kr (J.Y.); tkdqls2052@konkuk.ac.kr (S.L.); 2 Department of Mechanical Engineering, Konkuk University, 120 Neungdong-ro, Gwangjin-gu, Seoul 05029, Republic of Korea

**Keywords:** coating defect, edge, precise edge detection, primary color, roll-to-roll system, visual feature

## Abstract

Roll-to-roll (R2R) manufacturing depends on a system’s capability to deposit high-quality coatings with precise thickness, width, and uniformity. Therefore, consistent maintenance requires the immediate and accurate detection of coating defects. This study proposes a primary color selection (PCS) method to detect edge defects in R2R systems. This method addresses challenges associated with training data demands, complexity, and defect adaptability through a vision data-centric approach, ensuring precise edge coating defect detection. Using color information, high accuracy was achieved while minimizing data capacity requirements and processing time. Precise edge detection was facilitated by accurately distinguishing coated and noncoated regions by selecting the primary color channel based on color variability. The PCS method achieved superior accuracy (95.8%), outperforming the traditional weighted sum method (78.3%). This method is suitable for real-time detection in manufacturing systems and mitigates edge coating defects, thus facilitating quality control and production optimization.

## 1. Introduction

Roll-to-roll (R2R) systems have emerged as promising technologies for high-speed, large-scale production across various areas, including flexible electronics, sensors, and electric vehicle batteries [1,2,3,4]. These systems provide continuous manufacturing processes, offering advantages such as increased production efficiency, reduced material waste, and enhanced product quality and consistency [5,6]. In an R2R setup, materials are continuously fed into the system as rolls, enabling rapid production as the manufacturing process occurs on the moving material web [7,8,9]. A key process of R2R manufacturing is the coating process, where a thin layer of material is applied to a substrate surface to impart specific functionalities or properties [10,11]. As coating quality significantly influences the deposition of conductive materials, insulating materials, functional layers, consistent monitoring, and maintenance of the process are required [12]. The success of R2R manufacturing relies on the capability of the system to deposit high-quality coatings with precise thickness, width, and uniformity across a large area [13]. Therefore, the immediate and accurate detection of coating defects using vision images is necessary.

**Figure 1:**
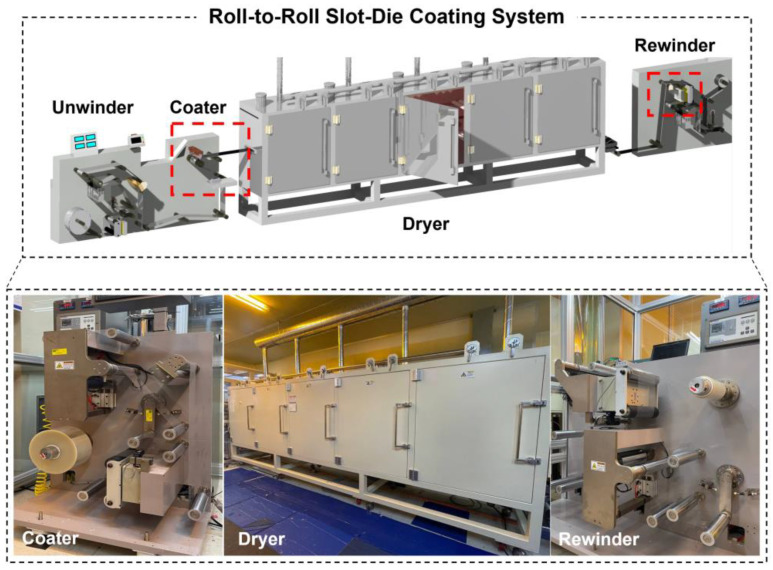
Roll-to-roll (R2R) slot-die coating system.

R2R manufacturing processes involve multiple approaches for coating materials, including gravure printing, slot-die coating, screen printing, and spray coating [14]. Each method possesses distinct advantages and limitations based on material properties, desired coating thickness, and production volume [15]. Among these approaches, the slot-die coating process achieves precise and uniform coating thickness and width over large areas, ensuring high material efficiency and minimal waste. A slot-die coating system is shown in Figure 1. Owing to its unique slot-shaped delivery system and adjustable coating parameters, this system effectively controls the thickness, uniformity, and material utilization of the product [16]. However, despite these advantages, a common coating defect known as the edge defect frequently occurs during the coating process.

**Figure 2:**
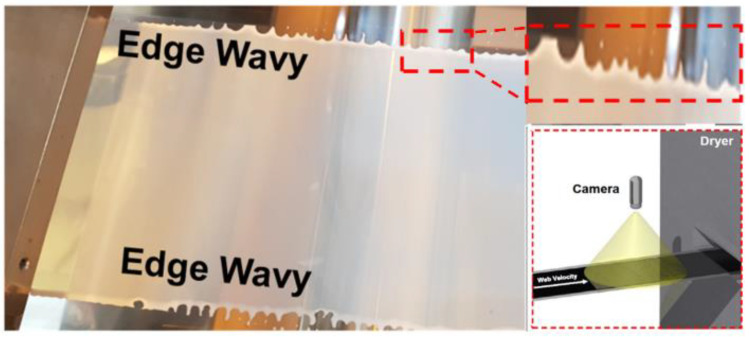
Examples of edge defects in an R2R manufacturing system.

Edge defects (Figure 2) occur as wave-like patterns at the edges of the coated material and can be attributed to various factors, including an uneven flow rate, substrate tension, coating thickness, and viscosity [17]. An uneven coating material flow can create a ripple effect, resulting in wave-like patterns along the edges. Similarly, a nonuniform substrate tension or inconsistent coating thickness can lead to edge defects [18]. Additionally, the viscosity of the coating material can contribute to this defect; a significantly high or low coating viscosity can lead to uneven coating and the formation of edge defects.

Defects can occur during the coating process owing to various factors. Therefore, timely detection and identification of these defects are crucial for their immediate elimination during R2R manufacturing [19,20]. Furthermore, despite the benefits of slot-die coating in R2R manufacturing, detecting coating defects during the process remains challenging. Current inspection procedures often rely on manual inspections or offline sampling, which can be time-consuming and ineffective for fault detection. Consequently, a vision-based system capable of the real-time detection of coating faults during the slot-die coating process is required. Such a system can significantly enhance quality control measures and help minimize production costs.

This study employs the primary color selection (PCS) method as a vision data-centric approach to detect edge defects in R2R slot-die coaters during the coating process. The proposed PCS method operates as an image-based technique for feature selection, thereby enhancing defect detection accuracy while minimizing the required data capacity for diagnostics. Given that the ultimate objective of a vision-based system for detecting coating defects is real-time operation, extracting relevant and valid features becomes crucial. The presence of invalid and miscellaneous features can compromise both detection accuracy and processing speed. Therefore, the PCS method is essential for effective monitoring and timely detection of coating defects.

Existing goal-oriented diagnosis and prognosis approaches in certain applications often overlook the performance of diagnostic and prognostic models. Recent studies have highlighted the significance of training time and data requirements in addition to accuracy [19,20,21,22,23]. Identifying relevant features that can enhance diagnostic accuracy, reduce processing time, and optimize data capacity for real-time monitoring is crucial [5,24]. However, current research primarily focuses on quantitative data, such as tension and vibration, when examining the features influencing multiple performance factors. In this study, we propose the PCS method, which expands the consideration of AI model performance factors and applies them to vision image data.

Studies focusing on vision image-based defect detection methods have primarily focused on the structure of deep neural networks to facilitate efficient learning [25,26,27,28,29]. Many of these studies have proposed modifications to learning algorithms or structural layers within neural networks to achieve high-accuracy detection [30,31,32,33,34]. However, the proposed PCS method selects the input data for utilization by the algorithm or model. Sun et al. [35] investigated and proposed effective defect detection methods based on machine vision data, achieving a defect detection accuracy rate of 99%. Similarly, Yan et al. [36] employed computer vision for in-line defect detection using deep learning methods. Although these studies provided promising results, many faced challenges such as the requirement of extensive training data, computational complexity, or limited adaptability to different defect types [37,38,39]. The PCS method proposed in this study addresses these challenges through a vision data-centric approach, ensuring efficient feature selection for the accurate detection of edge coating defects in R2R slot-die coaters.

The PCS method achieved an average detection accuracy of 95.8%, surpassing the 78.3% achieved by the weighted sum method. Moreover, the processing time and data capacity required for the PCS method were significantly lower compared to those of the weighted sum method, rendering it a practical and efficient approach for real-time defect detection in R2R manufacturing systems.

## 2. Materials and Methods

### 2.1. Vision Data-Centric Edge Wave Coating Defect Detection Process

**Figure 3:**
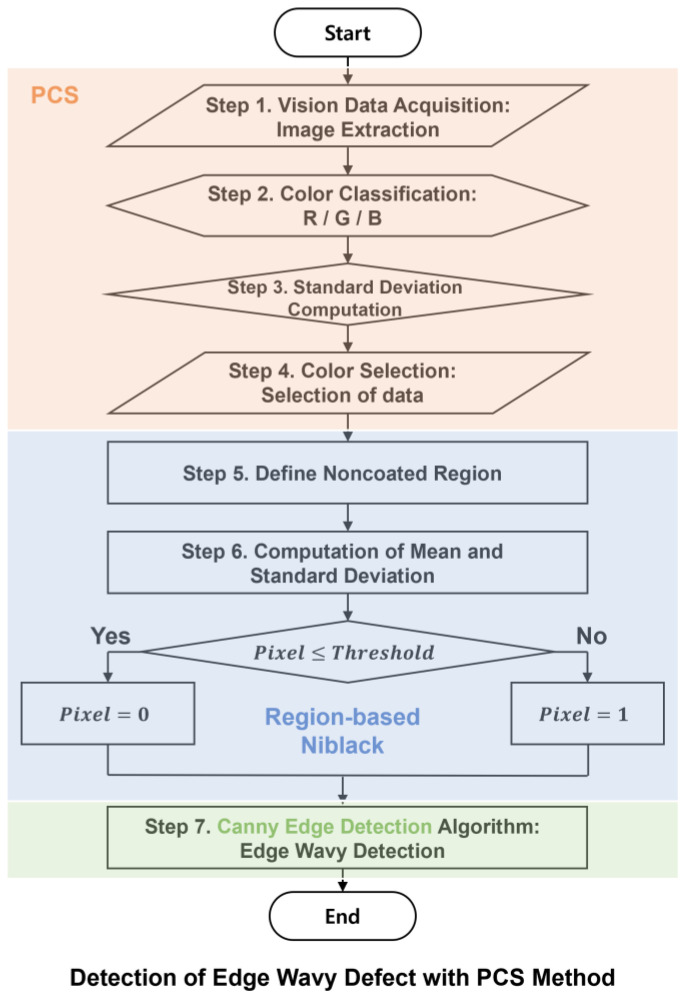
Flowchart of edge defect detection using the proposed method.

The edge defect detection process employs three distinct methods: PCS, region-based Niblack (RN) thresholding, and Canny edge detection. Figure 3 shows each method using a different color. This process comprises seven specific sequential steps to identify edge coating defects. The flowchart in Figure 3 illustrates the defect detection procedure. The PCS method, covering steps 1–4, entails processing vision image data for data selection. RN thresholding, performed during steps 5 and 6, determines the threshold value for classifying pixels as either white or black. The final step, step 7, involves Canny edge detection to identify edge patterns in the processed images. Steps 1–4 constitute the core process of the proposed approach presented in this study. 

The design theory of PCS involves a vision data-centric approach for effective edge coating defect detection in R2R slot-die coaters. The PCS method employs a unique color selection strategy based on the standard deviation of the red, green, and blue color channels. It intricately incorporates key principles: Vision cameras capture real-time images under specific processing conditions, which are then separated into red, green, and blue colors for subsequent analysis. The standard deviation of each color image is systematically calculated to quantify the variation between pixels, playing a pivotal role in gauging color variability within the image. Leveraging the calculated standard deviation values, the PCS method strategically selects a specific color channel deemed optimal for defect detection, enhancing its ability to discriminate between coated and noncoated regions. These design principles collectively define the theoretical foundations of the PCS method, emphasizing color variability as a key determinant in achieving precise edge defect detection. Through the experimental setup under the processing conditions mentioned in Section 3, the results highlight the percentage of the actual edge areas, areas detected using the weighted sum method, and edge areas identified using the PCS method. Detection accuracy is calculated using the equation provided in the manuscript. The PCS method ensures accurate edge detection and minimizes data capacity requirements. A detailed description of each step is provided below:

Step 1: The data acquisition process involves using computer vision to collect image data. This is achieved by installing vision cameras to capture real-time images under specific processing conditions.

Step 2: The acquired images are separated into red, green, and blue colors. Then, each image undergoes further separation into three distinct color images.

Step 3: The standard deviation of each color image from Step 2 is analyzed to determine the variation between pixels.

Step 4: Based on the calculated standard deviation value from Step 3, a specific color is selected and used as the data for defect detection.

Step 5: The RN method targets and focuses on the noncoated region to determine the threshold value.

Step 6: The threshold value is determined by evaluating the mean and standard deviation of the pixels in the noncoated region. Based on the result, the pixel values are classified as binary values “0” or “1,” representing black or white, respectively.

Step 7: Edge detection is performed based on Canny edge detection.

### 2.2. PCS Method

The PCS method represents an image processing approach designed to enhance the accuracy of coating defect detection while minimizing time and data requirements. Unlike a general image processing method that uses the weighted sum of RGB values to convert grayscales, as indicated in (1), this weighted sum method has limitations in accurately differentiating between coated and noncoated areas, particularly in cases with ambiguously colored regions. Moreover, this method leads to an unnecessary increase in data size and includes uncertain indicators, consequently lowering the detection accuracy. By contrast, the proposed PCS method adopts a specific color based on the standard deviation to identify the optimal color for distinguishing between coated and noncoated regions. The grayscale vector in the PCS method is obtained using (2).

*Grayscale of the Weighted Sum* = *R* × 0.30 + *G* × 0.59 + *B* × 0.11 (1)

*Grayscale of PCS* = *max*((*std*(*R*), *std*(*G*), *std*(*B*)) (2)

**Figure 4:**
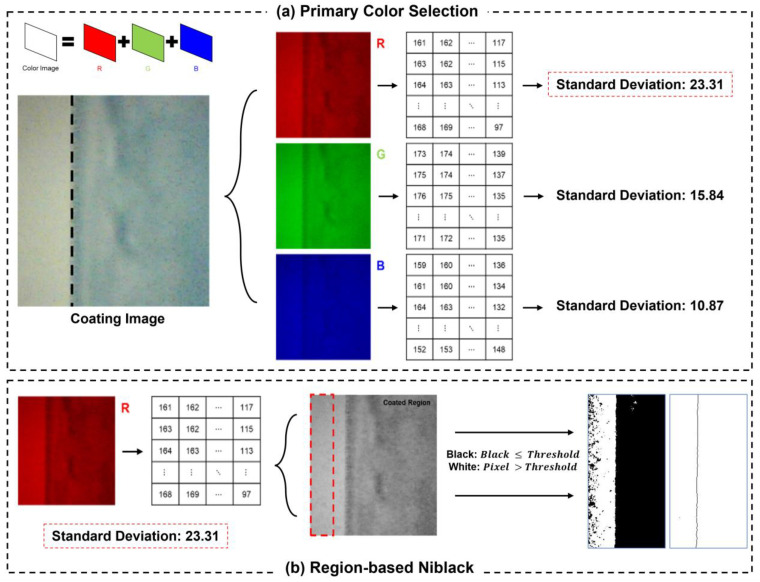
Schematic of the PCS and RN methods.

A schematic of the PCS method is shown in Figure 4. This diagram outlines the sequential steps. After capturing the coated image through experimental acquisition, it was separated into visual features of red, green, and blue image components. Each pixel within these color images was assigned a value between 1 and 255, representing the image brightness. These numerical values are directly proportional to the brightness levels, and they are employed to calculate the standard deviation using (3). A higher standard deviation value indicates a significant difference in average values among the pixels, implying that as the deviation increases, the differentiation between coated and uncoated areas becomes easier. This differentiation is crucial for effective edge detection, and PCS is applied to facilitate it. In other words, a larger deviation enhances detection performance by effectively distinguishing between the regions. After obtaining the standard deviation values for red, green, and blue, the color with the highest standard deviation is selected to proceed to the next step in the overall process. Considering the example shown in Figure 4, red is chosen, as it exhibits the highest standard deviation. Therefore, it is further processed using the RN method.

(3) $$\mathrm{Standard}\;\mathrm{Deviation}=\sqrt{\frac{\sum {\left(x-\bar{x}\right)}^{2}}{n-1}},$$

where x denotes the pixel and n represents the number of pixels.

The image features studied in this research primarily revolve around the color characteristics extracted through the PCS method for detecting edge coating defects. The PCS method focuses on the standard deviation of individual color channels (red, green, and blue) to select the most informative color for defect detection.

As the PCS method aims to enhance the accuracy of the coating defect detection based on the standard deviation of each color, there are factors that influence the accuracy of the PCS method, such as image quality and resolution, color variability, lighting conditions, coating material characteristics, environmental factors, algorithm parameters, and edge detection algorithm sensitivity. For image quality and resolution, the quality and resolution of the input images play a pivotal role in the ability of the PCS method to discern between coated and noncoated regions. Higher image quality and resolution contribute to more precise defect detection. Moreover, the inherent color variability present in both the coating material and substrate significantly influences the PCS method. Changes in lighting conditions during image acquisition can also affect the extraction of color information by the PCS method. Different coating materials can also exhibit distinct color properties. As there are numerous factors that can affect the detection accuracy of the PCS method, the edge defect detection process must be optimized, as shown in Figure 3. 

### 2.3. Region-Based Niblack Method

The Niblack method is a color binarization technique used to classify colors into either black or white based on a threshold value. The RN method focuses on a specific image region to determine an optimal threshold value. In this study, the RN method identifies the noncoated area as the targeted region. The noncoated area is selected because the coated area often exhibits a mixture of various shades and intensities, which makes setting a precise threshold challenging. By establishing the threshold based on the relatively uniform color of the noncoated area, the pixels can be accurately classified into either black or white.

A similar approach to the RN method is Otsu’s method, which addresses the presence of outliers in each region. These outliers can disrupt threshold determination, as they aim to minimize the variance. Therefore, the RN method overcomes the potential interference caused by outliers in each region during threshold determination. An optimized threshold yields a clear boundary, significantly enhancing the effectiveness of the Canny edge algorithm for defect detection. The threshold value of the RN method can be computed using (4), where NP represents the pixels in the noncoated region.

(4) $$\mathrm{Threshold}=\mathrm{mean}\left(\mathrm{NP}\right)-\mathrm{std}(\mathrm{NP})$$

### 2.4. Canny Edge Detection Algorithm

Various edge detection methods exist, such as the Sobel, Prewitt, and Robert Edge methods, which can be used for the detection of edge wave defects. Each method possesses unique characteristics and strengths. The Sobel method is particularly sensitive to diagonal edges and is resistant to noise. However, its effectiveness diminishes in densely or complexly changing contrast regions, making it less suitable for edge defect detection. Similarly, the Prewitt edge detection method shares similarities with the Sobel method but boasts a faster response time. However, compared to the Canny edge method, the edges detected may be less prominent as it is less sensitive to changes in brightness. The Robert Edge method is the fastest among the considered edge detection methods and is capable of reliably extracting edges. However, the edges extracted tend to be thinner and more susceptible to noise compared to other methods. Conversely, the Canny edge algorithm is widely regarded as the most optimized algorithm for determining contours and removing edges associated with noise in the original image. This algorithm excels at extracting strong edges while being relatively insensitive to noise. Therefore, the Canny edge algorithm has been employed due to its ability to detect edges in images accurately while minimizing false positives [40,41,42].

**Figure 5:**
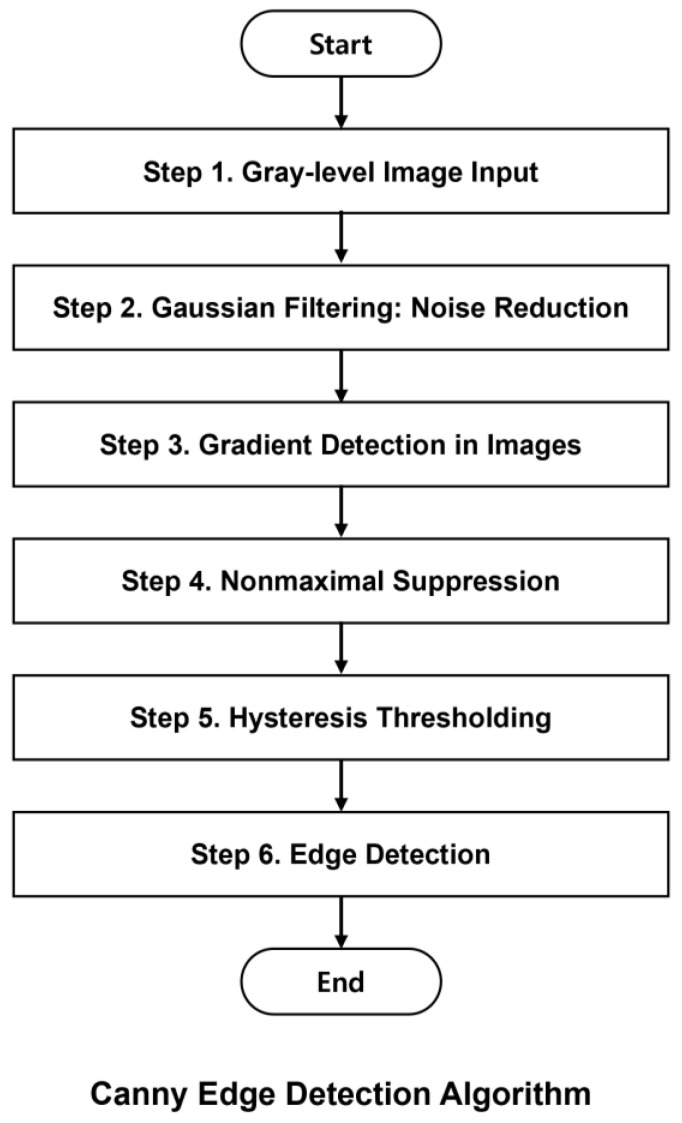
Flowchart of Canny edge detection.

Canny edge detection, widely used for accurately detecting edges in images, achieves reliable results through several steps. As shown in Figure 5, the gray-level image is the input, which then undergoes preprocessing via Gaussian filtering to reduce noise. Subsequently, the gradient is computed using edge detection operators. Non-maximum suppression is then applied to refine the detected edges, followed by hysteresis thresholding, which further enhances the edge map by classifying pixels as either strong or weak edges based on predefined thresholds. The algorithm produces thin and precise edge representations while minimizing false positives. Therefore, following the PCS and RN processes, the Canny edge detection algorithm identifies the edge coating defects in R2R slot-die coaters.

### 2.5. Computation of the Edge Wave Area

**Figure 6:**
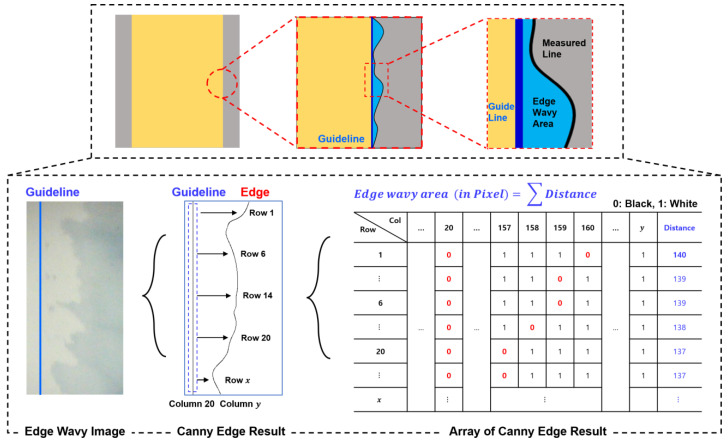
Edge wavy area computation.

The area of the edge wave is obtained by measuring the distance from the guideline to the edge (measured line) for each pixel row in the image. Subsequently, the sum of the calculated distances for each row is used to determine the area of the edge wave. This process is exemplified in Figure 6, where the first row shows a guideline in Column 20 and an edge (measured line) in Column 160. Measuring Row 1 values involves subtracting these two column numbers. The resulting computed area of the edge wave is used to verify the efficiency of the PCS method in comparison to the weighted sum method.

## 3. Experimental Design and Data Acquisition

**Figure 7:**
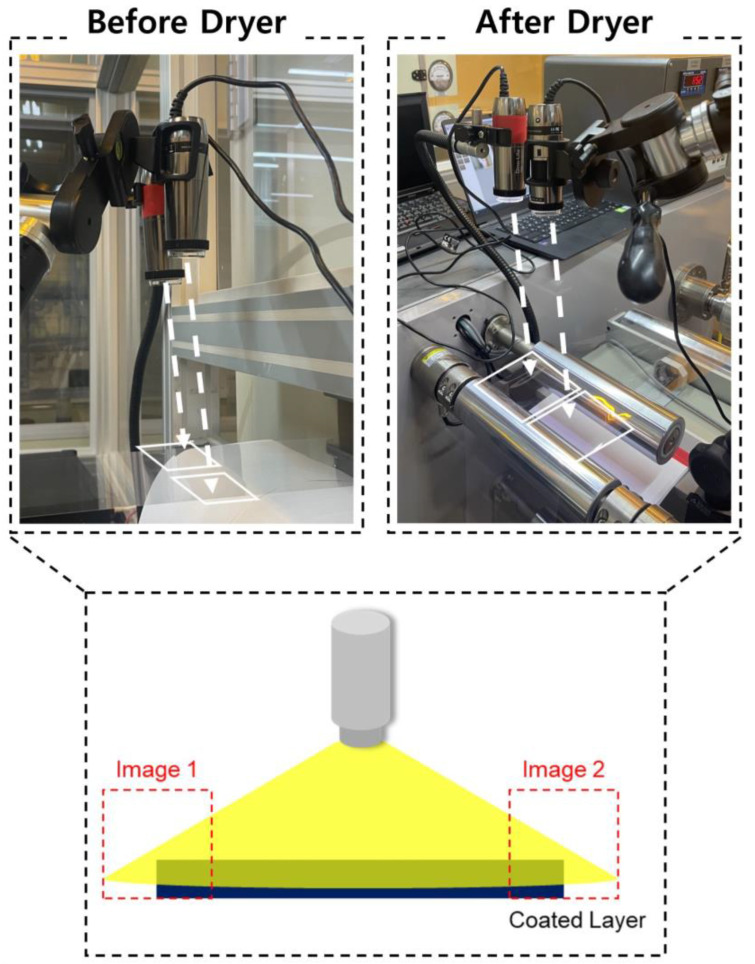
Vision camera setup of the R2R slot-die coater for edge detection.

**Figure 8:**
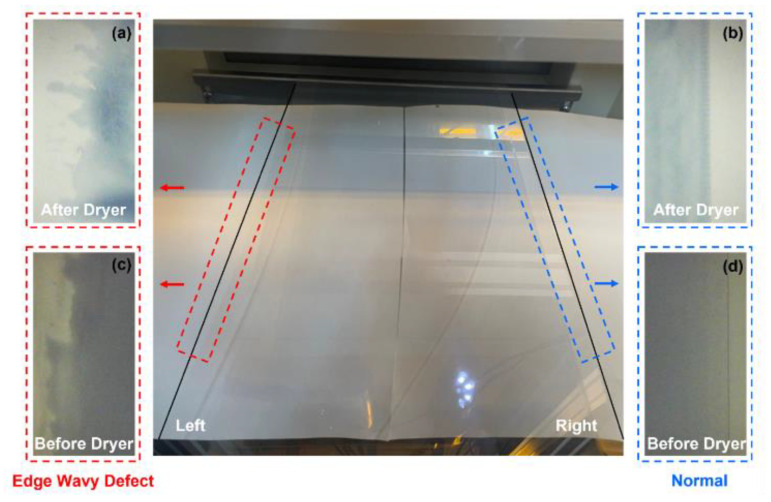
Comparison of the coating structural quality of coatings with and without edge defects. (**a**) Edge wave defect after dryer. (**b**) Edge wave defect before dryer. (**c**) Normal condition after dryer. (**d**) Normal condition before dryer.

The vision camera setup, based on the R2R slot-die coating system, is shown in Figure 7. Vision cameras (resolution: 640 × 480; magnification: 10×, frame rate: 60 fps) were strategically installed before and after the drying section, enabling real-time image acquisition. Figure 8 shows distinct variations in edge patterns and colors influenced by the drying section. “Normal” images are compared with edge defect images, highlighting significant coating quality differences. Four cameras captured images before and after drying. The PCS method was exclusively used to analyze post-drying images for defect detection. This study aims to demonstrate the advantages of the PCS method over the weighted sum approach.

The edge defect observed in this study is primarily caused by variations in coating thickness and uneven drying rates during the slot-die coating process. The interaction between the surface tension of the PEDOT:PSS ink, substrate tension, and coating viscosity influences the formation of edge defects on the PET film surface. The viscosity of the PEDOT:PSS ink plays a crucial role in determining its flow and leveling properties during the coating process. Higher viscosity can hinder the flow of the ink, leading to nonuniform coating thickness and the formation of edge defects. Similarly, variations in surface tension can affect the wetting behavior of the ink on the substrate surface, influencing the dduniformity of the coating application. Understanding the mechanical properties and behaviors of these materials, particularly in the context of edge defect formation, is crucial for optimizing coating processes and improving product quality. 

As shown in Table 1, information about the PET film, including its physical properties and surface characteristics, will enhance the understanding of the interactions between the substrate and the PEDOT:PSS ink. The compatibility between the substrate and ink can impact the adhesion and uniformity of the coating, thereby influencing the occurrence of edge defects. By including a detailed analysis of these material properties, we aim to provide a comprehensive understanding of the factors contributing to edge defect formation and their implications for polymer science and manufacturing processes.

**Figure 9:**
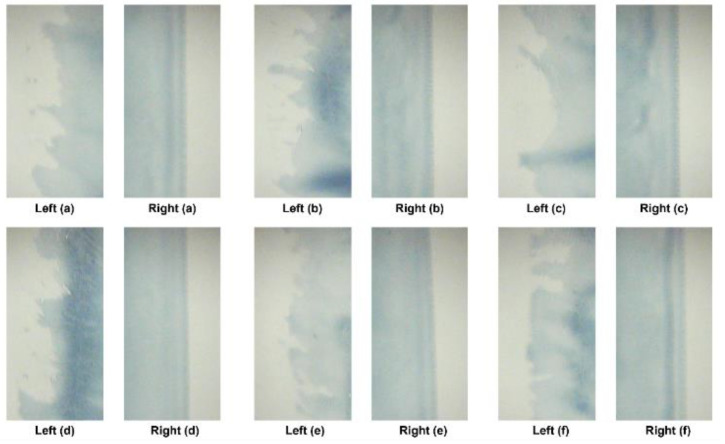
Vision images captured after drying: edge defects (**left**) and normal conditions (**right**). (**a**–**f**) Defect and Normal conditions for after dryer.

**Figure 10:**
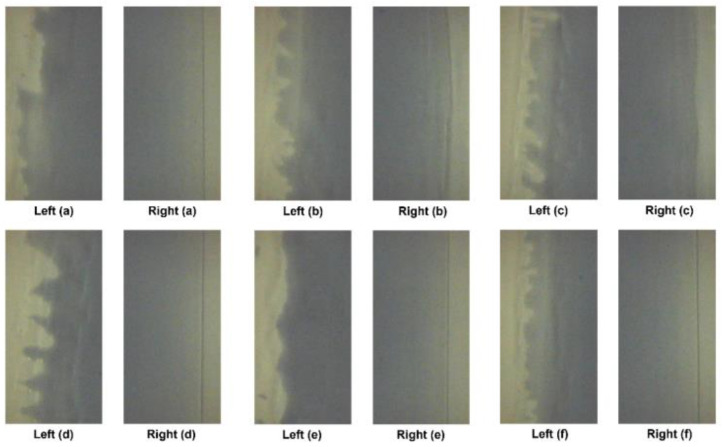
Vision images captured before drying: edge defects (**left**) and normal conditions (**right**). (**a**–**f**) Defect and Normal conditions for before dryer.

The processing conditions for data acquisition and the specific ink and material that were used during experimentation are listed in Table 1 and Table 2. The coating gap of the processing condition was 0.1 mm, and the flow rate was 4 mL/min. The drying temperature of the dryer was set to 80 °C, while the web speed of the R2R slot-die coating system was set at a speed of 1 m/min. The processing condition of the web tension was 2.7 kgf, the target thickness was 3 mm, and the coating width was 0.12 m. Coating was performed on the web for 20 min. The vision images of the experimental data were acquired over 20 min for 10 trials. The film that was used for acquiring vision images was CH34P. The poly(3,4-ethylenedioxythiophene):polystyrene sulfonate (PEDOT:PSS) + ethanol ink, known for its high working function and conductivity, offers remarkable transmittance and thermal stability. This is extensively applied as an electrode in flexible polymer solar cells and similar devices. Figure 9 and Figure 10 show examples of the images acquired under the processing conditions presented in Table 2. The images labeled “Right” depict coatings with nondefective edges, serving as a reference for direct comparison with coatings exhibiting edge defects. As mentioned earlier, the PCS method was applied to the “After Dryer” samples for comparison with results from the weighted sum method.

The images of the “After Dryer” cases were processed for standard deviation calculations to determine the primary color of the image for visual feature selection. As listed in Table 3, each image was extracted in red, green, and blue to evaluate the highest standard deviation for each case. As mentioned in Section 2, the highest standard deviation was used to determine the optimal color for distinguishing the coated and noncoated regions. A higher standard deviation value indicated a significant difference in average values among the pixels, implying that as the deviation increased, the differentiation between coated and uncoated areas became easier. The results show that red was the highest in Cases 1 and 5, green in Cases 3 and 6, and blue for Cases 2 and 4. The chosen color for each case was then processed through the RN and Canny edge detection methods. 

## 4. Results and Discussions

The edge coating defect detection results in the R2R slot-die coating system, following the procedures described in Section 2, are listed in Table 4 and Table 5. These tables display defect detection results for six edge vision images, processed using the methods outlined in Figure 3. The results of the proposed PCS method are shown alongside those obtained using the weighted sum method for comparison. These results include the percentages of actual edge wave areas, areas detected based on the weighted sum method, and edge areas identified using the PCS method. These percentages contribute to calculating the edge detection accuracy using (5). Furthermore, Table 4 and Table 5 present the processing time and data capacity required to compare PCS and weighted sum defect detection results.

(5) $$\mathrm{Accuracy}=100-/\left(\mathrm{Actual}\;\mathrm{Edge}\;\mathrm{Wave}\;\mathrm{Area}-\mathrm{Measured}\;\mathrm{Edge}\;\mathrm{Wave}\;\mathrm{Area}\right)\left[\%\right]/$$

The actual edge area mentioned in (5) was manually determined to assess the detection accuracy of both the PCS and weighted sum methods and validate the results obtained using the two compared methods. The edge wave area obtained using (5) was measured using the methods described in Section 2.

**Figure 11:**
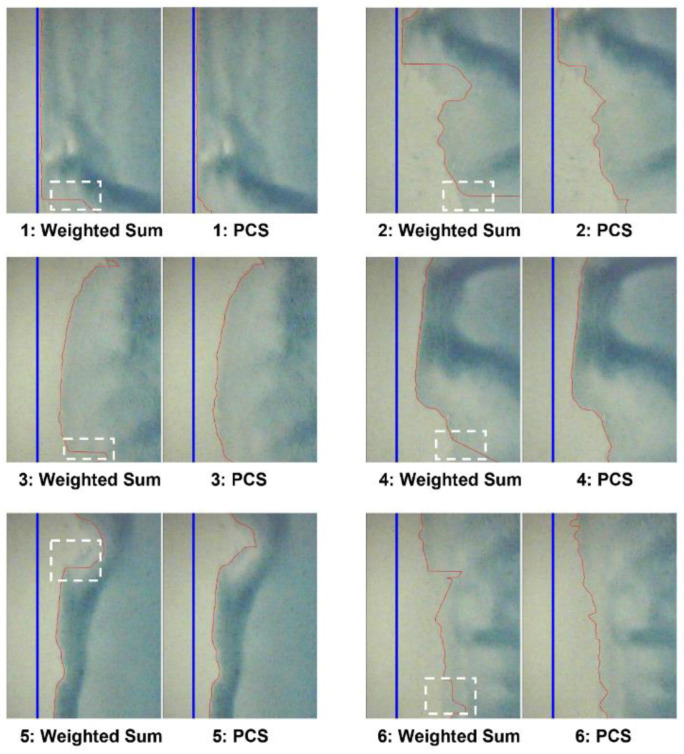
Comparison of edge detection results: weighted sum vs. PCS.

A comparison between the results of the weighted sum and PCS methods revealed that the PCS method enhanced the defect detection accuracy by approximately 12.1%. Furthermore, the required data capacity for defect detection decreased by approximately 33.33% on average. Additionally, the processing time significantly decreased from 147.8 s to 36.8 s, representing a decrease of approximately 75.1% using the PCS method. Therefore, the advantages of the PCS method are quantitatively evident and shown in Figure 11. 

As shown in Figure 11, the blue line indicates the guideline, representing the edge of the film, while the red line represents the measured line that distinguishes the coated and noncoated areas. The white dashed rectangles within the figure indicate the edge wave regions that are not detectable using the weighted sum method. 

**Figure 12:**
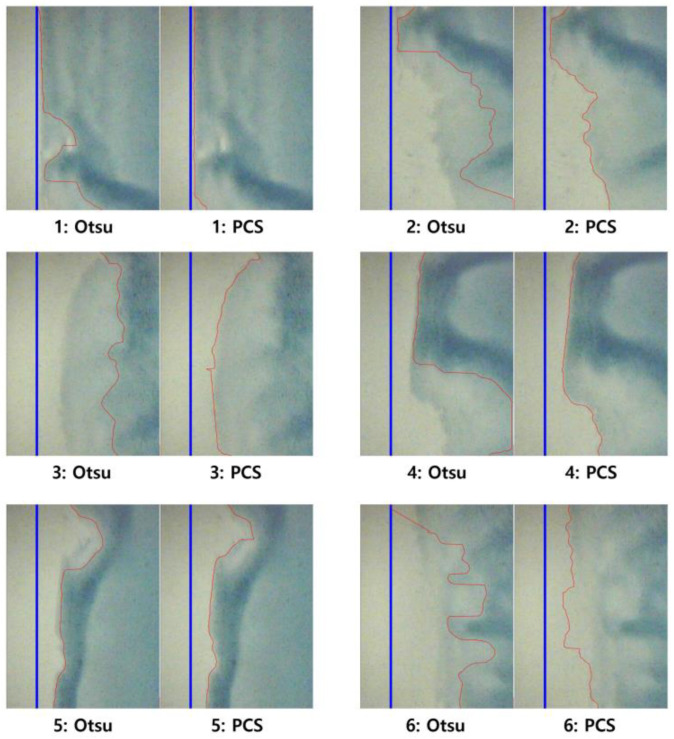
Comparison of edge detection results: Otsu’s method vs. PCS.

Although the primary focus of the study is to contrast the PCS method with the traditional weighted sum method, an additional experiment was conducted to compare the PCS method with another grayscaling method for verification. Otsu’s method was used as an alternative edge defect detection method in comparison to the PCS method [43,44,45]. The results of this extended comparison are listed in Table 6, alongside the results of the PCS and weighted sum methods. The results reveal an average of 80.7% accuracy for detecting edge areas while performing at a slower processing speed. Otsu’s method shows inconsistent accuracy among the six cases, ranging from 68.2% to 94.2% depending on the condition of the targeted image. Compared to the weighted sum method, even though it shows better performance in terms of time and data capacity consumption, the inconsistency of this method critically affects the defect detection process. The inconsistent results of measuring the edge wave area are shown in Figure 12. This broader evaluation not only highlights the superior performance of the PCS method but also highlights its advantages in edge coating defect detection compared to other prevalent image preprocessing techniques.

The observed variation in accuracy between Otsu’s method and the PCS method can be attributed to differences in their underlying principles and implementation strategies. Otsu’s method is a thresholding technique widely used for image segmentation tasks, relying on the calculation of an optimal threshold value to separate foreground and background pixels in an image. However, the performance of Otsu’s method can vary depending on factors such as noise levels, lighting conditions, and the presence of outliers, leading to fluctuations in accuracy. 

In contrast, the PCS method introduced in this study offers a specialized approach for edge defect detection in R2R slot-die coating systems. By leveraging primary color selection based on standard deviation, the PCS method aims to identify the optimal color channel for defect detection while minimizing data capacity requirements and processing time. This tailored approach enables the PCS method to accurately distinguish between coated and noncoated regions, enhancing defect detection accuracy in industrial settings. 

In the experiments, the PCS method consistently outperformed Otsu’s method in terms of accuracy, highlighting its effectiveness for targeted defect detection tasks in R2R coating systems. While Otsu’s method provides a general thresholding approach, the PCS method offers a specialized solution optimized for the challenges associated with coating defect detection. In accuracy, the PCS method surpassed Otsu’s method by 17.3% on average. Furthermore, the PCS method consumes less processing time by 60.7% than Otsu’s method. Therefore, the observed variation in accuracy underscores the importance of employing specialized techniques like the PCS method for enhancing defect detection performance in industrial applications. 

**Figure 13:**
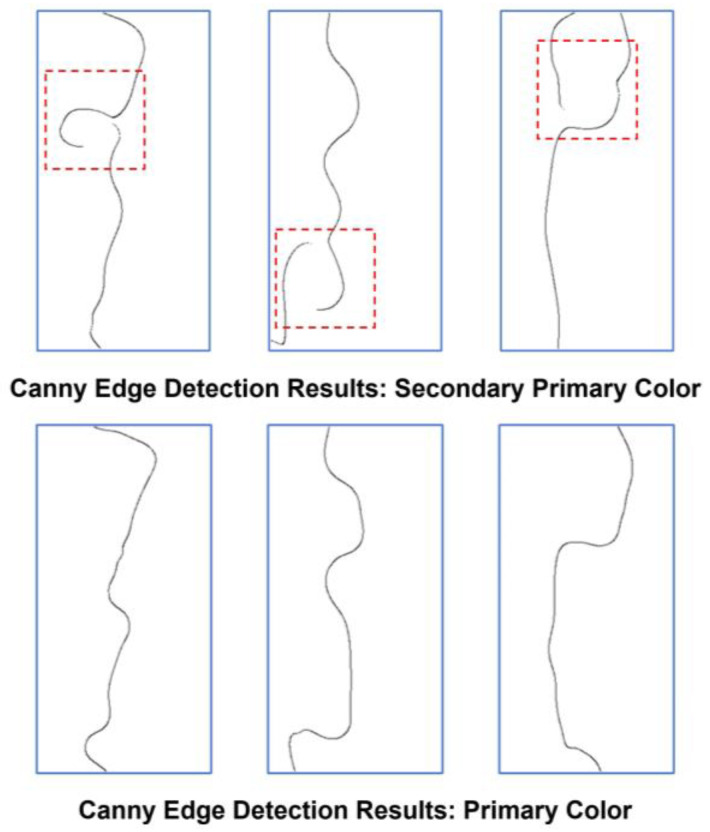
Comparison of Canny edge detection result based on primary and secondary color.

The basis of selecting the optimal color with the PCS method is to derive a higher detection rate than other approaches. The significance of this selection lies not only in the potential reduction of processing time and data capacity but predominantly in the enhancement of detection capabilities. However, opting for secondary or tertiary primary colors may reduce processing time and data capacity, but it may not yield the same increase in detection rate as with primary colors. As shown in Figure 13, the selection of secondary primary color causes the lack of precision and clarity for the Canny edge detection algorithm to differentiate regions. Therefore, once the Canny edge detection algorithm is carried out based on the results of the secondary or tertiary primary color, the outcome of the edge defect detection rate will significantly decrease compared to the primary color results. The results of the defect detection based on secondary and tertiary primary color are shown in Table 7 and Table 8. The priority of the colors aligns with the result of the calculation of standard deviation as mentioned above. The results of secondary and tertiary primary colors show that while maintaining a similar reduction rate compared to the primary color, the accuracy of the edge defect has decreased. It can be seen that the average accuracy of the tertiary primary color is lower than the weighted sum average. Thus, resulting in the optimal primary color is crucial in order to maximize the significant efficiency the proposed PCS method derives. 

After verifying defect detection with the PCS method, several conditions exist that can be optimized for stabilizing the coating quality and its maintenance in R2R slot-die coating systems. Feedback mechanisms can be integrated into the manufacturing process to automatically adjust coating parameters when edge defects are detected. These adjustments may include real-time modifications of flow rates, substrate tension, or coating thickness to mitigate the occurrence of edge defects and ensure consistent coating quality. Through the flow rate adjustment, the flow rate of the coating material can be dynamically regulated in response to the PCS-detected defects. An automated control system can modify the flow rate to ensure a more uniform and controlled distribution of the coating material, mitigating the risk of uneven flow and subsequent edge defects. Substrate tension also plays a crucial role in coating quality. In the event of edge defects identified by PCS, the tension of the substrate can be adjusted in real time. This helps maintain a consistent substrate surface, reducing the likelihood of defects caused by tension variations. Additionally, the coating thickness is another critical parameter affecting the product quality. When PCS detects defects, adjustments can be made to modify the coating thickness. Automated systems can be used to achieve the desired specifications and minimize the impact of detected defects on the final product. By optimizing these conditions, an automated feedback control system can be implemented to enhance the stability and optimization of coating quality in R2R manufacturing.

In summary, the PCS method not only improves defect detection accuracy through color selection but also simultaneously reduces processing time and data requirements. Furthermore, selecting specific regions as references to determine color thresholds improved the accuracy of defect detection. While deep learning-based methods are renowned for their capability to learn features from data automatically, the PCS method is designed to operate efficiently with minimal data requirements. Its strength lies in its vision data-centric approach, which enables high defect detection accuracy while minimizing the need for extensive training data. Therefore, while defect detection can be achieved using deep learning, the PCS method’s primary goal is not only to detect defects accurately but also to achieve this using less time and data capacity than other methods.

This study provides a valuable contribution to the existing literature by introducing a new method based on computer vision and image processing for detecting defects in coatings applied using R2R slot-die coating machines. Previous research on the characteristics of various performance factors has primarily focused on quantitative data such as tension, vibration, and existing defect detection methods. However, these studies encounter challenges such as the need for large training datasets, computational complexity, or limited adaptability to different defect types. In contrast, the proposed PCS method serves as an image-based feature selection technique that enhances defect detection accuracy while minimizing the data capacity required for diagnostic purposes.

The novelty of this study lies in the development and application of the PCS method for the vision-based detection of edge wave coating defects in R2R slot-die coaters. The key elements that contribute to its originality are as follows: The study introduces the PCS method as a novel vision data-centric approach for coating defect detection. Unlike traditional methods, PCS focuses on color selection based on the standard deviation, ensuring optimal differentiation between coated and noncoated regions. The primary focus of this study is on addressing the specific challenge of edge wave coating defects in R2R slot-die coaters. The proposed method is tailored to detect these defects in real time during the manufacturing process. The PCS method contributes to the field by providing an efficient feature selection technique for defect detection. By emphasizing color variability and selecting the color channel with the highest standard deviation, the method optimizes the use of vision image data. This study systematically compares the performance of the PCS method with traditional weighted sum methods, demonstrating its superior defect detection accuracy, reduced processing time, and lower data capacity requirements. This method is designed considering the practical requirements of R2R manufacturing systems. Its real-time defect detection capabilities make it valuable for quality control and production optimization in large-scale, high-speed manufacturing processes.

## 5. Conclusions

This study introduced the PCS method as a vision-based approach for detecting edge coating defects in R2R slot-die coaters. This method effectively improved defect detection accuracy while minimizing data capacity requirements and processing time. By selecting the color with the highest standard deviation as the basis for defect detection, the PCS method could accurately differentiate between coated and noncoated regions that are crucial for precise edge detection. The PCS method was further enhanced by combining the RN method and Canny edge detection algorithm to obtain precise edge representations and refine defect detection. 

Experimental results demonstrated that the PCS method outperformed the traditional weighted sum method in terms of defect detection accuracy. The PCS method achieved an average detection accuracy of 95.8%, whereas the weighted sum method achieved 78.3%. Additionally, it also showed robustness in handling various edge patterns influenced by different processing conditions, as evidenced by consistent and reliable defect detection results across different vision images. Furthermore, the PCS method exhibited significantly lower processing time and data capacity requirements compared to the weighted sum method, making it a practical and efficient approach for real-time defect detection in R2R manufacturing systems.

This study contributes to the field of R2R manufacturing by addressing the challenge of edge coating defects in slot-die coaters. The PCS method overcomes the limitations of extensive training data requirements, computational complexity, and limited adaptability to different defect types using a vision data-centric approach. It ensures efficient feature selection for accurate edge coating defect detection in R2R slot-die coaters while minimizing the required data capacity for diagnostic purposes. The findings of this study have practical implications in industrial applications, where real-time defect detection is crucial for ensuring product quality and reducing production costs. 

While the introduced methods, including the PCS method, primarily focus on enhancing the detection of edge coating defects rather than directly reducing them, their implementation can lead to significant savings in the production process. The PCS method offers several advantages that contribute to improved efficiency and cost savings. 

Firstly, by enhancing defect detection accuracy, the PCS method enables early identification of coating defects, allowing for prompt corrective actions to be taken. This proactive approach helps minimize material wastage and rework, reducing production downtime and associated costs. Additionally, by accurately identifying edge defects, the PCS method facilitates targeted interventions to address underlying process issues, such as substrate tension or coating thickness variations, thereby preventing the recurrence of defects in subsequent production runs.

Furthermore, the PCS method’s ability to minimize data capacity requirements and processing time translates to improved throughput and productivity in R2R manufacturing systems. With faster defect detection and analysis, production lines can operate more efficiently, leading to higher throughput and reduced cycle times. Moreover, the PCS method’s real-time defect detection capabilities enable immediate feedback adjustment of process parameters, ensuring consistent coating quality and reducing the likelihood of defective products reaching the market.

Overall, while the PCS method may not directly eliminate edge coating defects, its implementation can result in substantial savings in the production process by improving defect detection accuracy, enhancing operational efficiency, and reducing material wastage and rework costs.

As for the limitations of this study, indeed, the choice of substrate can influence the properties and performance of PEDOT:PSS coatings. Variations in substrate properties such as surface roughness, surface energy, and composition can affect the adhesion, morphology, and conductivity of the coated films. Therefore, it is essential to consider substrate–material interactions when applying the PCS method for defect detection. While the PCS method itself is applicable to various coating materials, including PEDOT:PSS, the effectiveness of defect detection may vary depending on substrate characteristics. Substrates with significantly different properties or surface conditions may present challenges in accurately detecting edge coating defects due to variations in coating behavior and defect manifestation. Additionally, the PCS method’s ability to precisely distinguish between coated and noncoated regions may be limited in cases where substrate properties interfere with color selection or image processing algorithms. Therefore, while the PCS method offers a versatile approach to defect detection, its efficacy may be influenced by substrate-related factors, highlighting the importance of considering substrate–material interactions and limitations in real-world applications.

Therefore, future research directions may involve further improving this method by exploring advanced image processing techniques and investigating its applicability to other types of coating defects and substrates. Additionally, the integration of the PCS method into an automated feedback control system could be explored to enable real-time defect correction during R2R manufacturing.

## Figures and Tables

**Table 1:** Material properties of PET film and PEDOT:PSS ink.

Material	Properties	Unit	Value
PEDOT:PSS + Ethanol (1:1)	Viscosity	cP	10
	Surface Tension	mN/m	25.14
PET (CH34P)	Thickness	μm	100
	Width	mm	0.25
	Elastic Modulus	MPa	2010
	Density	$kg/m^{3}$	1450
	Thermal Conductivity	W/(m·K)	0.290

**Table 2:** Processing conditions of data acquisition.

Ink and Film	Conditions	Unit	Value
Ink: PEDOT:PSS + Ethanol (1:1) Poly(3,4-ethylenedioxythiophene):Poly(4-styrenesulfonate) Film: PET (CH34P)	Coating Gap	mm	0.1
	Flow Rate	mL/min	4
	Drying Temperature	°C	80
	Web Speed	m/min	1
	Web Tension	kgf	2.7
	Target Thickness	mm	3
	Coating Width	m	0.12
	Camera Location	-	Before and After Dryer
	Coating Time	min	20
	Trial	-	10

**Table 3:** Primary color evaluation: edge images.

Standard Deviation of Edge Wave Images			
No.	Red	Green	Blue
1	24.01	17.56	19.04
2	19.47	18.15	22.54
3	16.75	19.20	18.47
4	20.32	17.44	21.85
5	23.90	17.21	18.96
6	17.50	19.85	16.60
Average	20.32	18.24	19.58

**Table 4:** Edge detection results: weighted sum method.

No.	Actual Edge Wave Area [%]	Weighted Sum			
		Measured Edge Wave Area [%]	Accuracy [%]	Processing Time [s]	Data Capacity [Mb]
1	1.1	14.8	86.3	144	6.13
2	21.7	36.5	85.2	152	6.01
3	19.4	32.1	87.3	147	6.08
4	19.0	32.2	86.8	145	5.72
5	16.2	30.4	85.8	148	6.17
6	22.7	38.3	84.4	151	5.94
Average	-	-	85.9	147.8	6.00

**Table 5:** Edge detection results: PCS method.

No.	Actual Edge Wavy Area [%]	Primary Color Selection			
		Measured Edge Wavy Area [%]	Accuracy [%]	Processing Time [s]	Data Capacity [Mb]
1	1.1	1.6	99.5	35	4.04
2	21.7	26.6	95.1	40	3.87
3	19.4	20.3	99.1	33	4.11
4	19.0	20.2	98.8	39	4.01
5	16.2	18.4	97.8	37	3.96
6	22.7	24.8	97.9	37	4.03
Average	-	-	98.0	36.8	4.00

**Table 6:** Edge detection results: Otsu’s method.

No.	Actual Edge Wave Area [%]	Otsu’s Method			
		Measured Edge Wave Area [%]	Accuracy [%]	Processing Time [s]	Data Capacity [Mb]
1	1.1	10.9	90.2	87	5.84
2	21.7	46.4	75.3	95	6.00
3	19.4	41.5	77.9	99	5.90
4	19.0	50.8	68.2	104	5.88
5	16.2	22.0	94.2	86	6.00
6	22.7	44.1	78.6	91	5.60
Average	-	-	80.7	93.7	5.87

**Table 7:** Edge detection results: secondary primary color.

No.	Actual Edge Wave Area [%]	Secondary Primary Color			
		Measured Edge Wave Area [%]	Accuracy [%]	Processing Time [s]	Data Capacity [Mb]
1	1.1	8.8	92.3	42	3.75
2	21.7	34.0	87.7	37	4.01
3	19.4	27.4	92.0	40	4.08
4	19.0	26.9	92.1	41	3.83
5	16.2	34.3	81.9	38	4.02
6	22.7	36.7	86.0	38	4.01
Average	-	-	88.7	39.3	3.95

**Table 8:** Edge detection results: tertiary primary color.

No.	Actual Edge Wave Area [%]	Tertiary Primary Color			
		Measured Edge Wave Area [%]	Accuracy [%]	Processing Time [s]	Data Capacity [Mb]
1	1.1	16.5	84.6	40	3.67
2	21.7	38.4	83.3	43	3.84
3	19.4	27.6	91.8	36	4.21
4	19.0	28.8	90.2	34	4.07
5	16.2	36.1	80.1	38	3.66
6	22.7	39.0	83.7	42	4.04
Average	-	-	85.6	38.8	3.91

## Data Availability

The original contributions presented in the study are included in the article, further inquiries can be directed to the corresponding authors.
